# A Study of Factors Contributing to Denture Stomatitis in a North Indian Community

**DOI:** 10.1155/2011/589064

**Published:** 2011-11-17

**Authors:** Amit Vinayak Naik, Ranjana C. Pai

**Affiliations:** Department of Prosthodontics, Teerthanker Mahaveer Dental College and Research Center, Delhi Road, Uttar Pradesh, Moradabad 244001, India

## Abstract

Factors like oral and denture hygiene, presence of saliva, age of the denture, and degree of colonization with *Candida albicans* are to be evaluated as local contributing factors for causing denture stomatitis. 100 patients aged 30 to 70 years were selected for the study. Among these, 70 patients were labeled test group showing signs of stomatitis and 30 patients as control group as they showed no inflammatory signs. Clinical tests included oral and denture hygiene evaluation, salivary measurements, and age of the dentures, and microscopic investigations were done. Results showed no significant differences between the two groups in terms of saliva, oral and denture hygiene habits, and denture age. Test group showed stomatitis in patients who were wearing dentures for 5 to 10 years compared to control group who were wearing dentures for 10 years and above. Denture age was proportional to *Candida* colonization and not to degree of inflammation. Significant differences were found in *Candida* colonization of the fitting surface of the denture between stomatitis and control groups. Poor denture hygiene habits are the most prominent contributing factor for denture stomatitis and colonization.

## 1. Introduction

Denture stomatitis also known as denture sore mouth and prosthetic stomatitis implies inflammation of oral mucosa especially palatal and gingival mucosa which is in direct contact with the denture base. The frequency of its development is 25 to 67% [1–4], mostly in women, and prevalence increases with age [5]. Clinically the inflammation is of varying degrees and classifications, Newton's classification being most commonly accepted [6].

Numerous studies have been done in the past to study the causes of the disease [1–27], but the main cause has not been agreed upon. Studies have pronounced different factors causing denture stomatitis like traumatic occlusion [5, 9], poor oral and denture hygiene [11, 14, 16–19], microbial factors [10–14], age of the denture [4, 16], allergy to the denture base materials [28], residual monomer [29], thermal stoppage below the denture [1–3], smoking, various types of irradiation, dryness of mouth [1, 2, 20], systemic conditions, diabetes mellitus and immunodeficiency [21], nutritional deficiencies [22], and medications [20, 23]. Plaque on the inner surface of the denture harbors microorganisms causing inflammation of the mucosa [2, 3, 17–19].

Hence, a study was designed to study the influence of the various local factors like saliva, oral and denture hygiene habits, age of the denture causing Candidal colonization.

## 2. Materials and Methods

A total of 100 subjects aged 30 to 70 years with a mean of 62 years (86 male and 14 female) were selected for the study. Patients with relined or rebased dentures were not included in the study. Again the subjects were divided into 2 groups: (a) *test group*: 70 subjects with inflammatory changes of the mucosa below the denture base; (b) *control group*: 30 subjects without inflammatory changes of the mucosa below the denture base. 

Case history sheets were prepared with questionnaire translated in the local language along with precise slots for the clinical findings of inflammation of oral mucosa. Quantity of saliva was measured by Quantum Q sal test [24]; measured quantities were marked by degrees: degree 0: normal salivation (>0.4 mL/min), degree 1: oligosialia (<0.4 mL/min), and degree 2: xerostomia (<0.2 mL/min). Oral hygiene evaluation was done by means of visual examination of plaque, dental calculus, and pigmentation quantity, after the use of plaque indicator. Oral hygiene was estimated by degrees: degree 0: poor oral hygiene, degree 1: satisfactory oral hygiene, and degree 2: good. Degrees of hygiene were also estimated for denture: degree 0: poor hygiene of the denture (over 1/3rd denture covered with plaque and calculus), degree 1: satisfactory (less than 1/3rd covered with plaque and calculus), and degree 2: good denture hygiene (without plaque and calculus). The age of the denture was also noted as shown in Table 1.

Swabs were taken from all the subjects from base of the denture for *Candida* albicans culture study. The acrylic base was slightly cut on the surface, and the remaining scraps soaked in the physiological salt solution were smeared by means of a sterile cotton stick onto nutritional Sabouraud dextrose agar substratum (Becton- Dickinson and Co, Cockeysville, USA, 25). Colonies of *Candida* albicans appeared after incubation for 48 hours in the thermostat at 37°C. Their number was expressed in degrees as explained by Olsen [26, 27].

Intensity of inflammation in the palate was estimated using the modified classification of Newton [6]: degree 1: poor intensity of focal inflammation with individual focal erythematous areas on the palatal mucosa, degree 2: marked inflammation on the entire palate, erythema affecting the palatal mucosa (below the base of the denture), and degree 3: marked inflammation accompanied by hyperplasia with papillary hyperplasia.

All variables in the test group were compared with the variables in the control group, and the data was analyzed. The significance of the differences was estimated by Chi-square test.

## 3. Results

Various factors were analysed as follows.

**Saliva**

Unstimulated saliva findings in both groups showed normal salivation in 55.3% subjects with denture stomatitis and 39% of the control group. There were more cases of xerostomia in control group (6.1%) as compared to test group (4.9%). Salivation quantum was not statistically different between the test and control groups (*P* = 2.5).

**Oral Hygiene**

More than 62% of both groups had satisfactory oral hygiene (*X* ^2^ = 0.6).

**Denture Hygiene**

Test group with denture stomatitis had poor denture hygiene (76%). No statistically significant difference was seen between both groups (*X* ^2^ = 0.8).

**Intensity of Inflammation**

Poor hygiene was seen to be directly proportional to intensity of inflammation. 84% of the subjects had class II inflammation (Newton), whereas only 9% had severe inflammation (class III) (*X* ^2^ = 0.1).

**Denture Age**

Test group showed most dentures to be in the range of 5 to 10 years old, whereas control group had dentures more than 10 years old. The difference in denture wear time was not statistically significant (*X* ^2^ = 0.3).

Denture age was not significantly responsible for the intensity of inflammation (*X* ^2^ = 0.1) as well as denture infection (*X* ^2^ = 0.9), although older dentures were more infected with *Candida* albicans in subjects with denture stomatitis (test group) as shown in Table 2.

**Contamination of the Denture**

*Candida* albicans was not found on the majority of the dentures in the control group (39%), whereas it was found on 54% of dentures of stomatitis patients, which was statistically significant (*X* ^2^ = 0.03) as shown in Table 3.

## 4. Discussion

The factors contributing to denture stomatitis have been shown to be varied and have interaction with local and systemic factors. Oral microorganisms change after wearing the denture, and this condition favors the growth of organisms causing denture stomatitis. *Candida* albicans [7, 8, 10–13] and bacterial interaction have shown to be prominent factors contributing to denture stomatitis. Newton's type I has been shown to be the result of trauma, whereas Newton's class III has multivariable interaction phenomenon [7–9]. 

Our study has evaluated different factors like saliva, denture age, and oral and denture hygiene, stating that there was hardly any statistically significant difference between the control group and test group showing that the disease cannot be solely caused by only a single local factor. Our study points out that xerostomia is not a very important factor in causing denture stomatitis as compared to previous studies which indicated otherwise [2, 5, 20, 23, 24]. Denture hygiene had a major role as compared to xerostomia.

Palatal inflammation has been shown to be more prominent in patients having poor denture hygiene but control group did not show inflammatory changes, which points out the importance of resistance of oral mucosa to be more important predisposing factor [14, 17, 19, 23]. 

Denture age is shown by previous studies to be an important factor as a result of poor fit, roughness, inadequate hygiene, and accumulation of plaque due to aging of denture [4, 16, 19]. In our study, it was seen that quality of denture was more important than the age of the denture [13, 15, 16, 19]. Highly finished and polished dentures had less chances to get contaminated as compared to old dentures which were maintained in a good condition. However, it could not be denied that aging of the denture and release of residual monomer with time results in poorer fit which affects the contamination of the denture. In our study, the contaminated dentures with *Candida* albicans had a very strong relation in contributing to denture stomatitis. The dentures in the control group were negligibly contaminated as compared to dentures of the test group.

Most of the subjects had type II (medium) inflammation which occurs due to the interaction of several factors among which, infection by *Candida* is the most important.

The integrity of mucosa is not necessarily threatened by age but may be a result of stress, trauma, disease, or drugs. Overall factors causing immunodeficiency weaken the resistance of mucosa making it susceptible to attack by bacteria, fungi, viruses, and parasites. Hence, it is shown that denture stomatitis results in the mouths of older people as an interaction of various local and systemic factors and solely denture wear cannot be taken as a cause of stomatitis when proper oral and denture hygiene methods are adopted.

## 5. Conclusion

1. There was no statistically significant difference in the local factors such as saliva, denture age, and hygiene between the test and control groups.
2. However, the degree of contamination of the denture by *Candida* albicans had a pronounced relation with the intensity of inflammation.
3. Poor oral and denture hygiene was determined as initial local factor predisposing to denture stomatitis.
4. Denture hygiene instructions, followup, and re-enforcement are very essential for the overall health of the oral cavity after denture rehabilitation.

## Figures and Tables

**Table 1:** Distribution of patients according to denture age.

Sign	Denture age	Test group	Control group
0	<5 years	10	4
1	5 to 10 years	33	9
2	>10 years	27	17

**Table 2:** Relation of denture age to inflammation.

	Degrees of inflammation		
	Class I	Class II	Class III
0-to-5-year-old denture	18%	5%	3%
5-to-10-year-old denture	20%	26%	0%
More-than-10-year-old denture	18%	14%	6%

**Table 3:** Comparison of *Candida* contamination on dentures in test group and control group.

	Degree of denture contamination by *Candida* albicans			
	0	1	2	3

Control group	38%	34%	6%	22%
Test group	14%	19%	14%	53%
